# Miniaturized antenna verified with diffuse optical measurements for native and boiled adipose tissue differentiation

**DOI:** 10.1038/s41598-022-19430-y

**Published:** 2022-09-03

**Authors:** Ashraf S. Abdel Halim, Mohanad Mostafa, Omnia Hamdy

**Affiliations:** 1 Department of Communication, Faculty of Engineering, Canadian International College (CIC), Cairo, Egypt; 2 Department of Engineering Applications of Lasers, National Institute of Laser Enhanced Sciences, Cairo University, Giza, Egypt

**Keywords:** Biomedical engineering, Electrical and electronic engineering

## Abstract

Medical industries are continuously working towards the development of wearable theragnostic devices which enable monitoring various ailments in the body and then transmitting them to the base-station. The antenna design is of prime importance where the suitable design guarantees proper communication between the antenna and the base-station. In this paper, a co-planar wave-guide antenna is proposed for the use in the medical implant communication service (MICS) band for data transmission. The proposed antenna is studied for ex-vivo applications where the antenna is simulated for bovine intramuscular fat (adipose tissue). The preliminary results showed that the antenna radiates in MICS band. Two types of samples are tested; namely, native fat and boiled fat. The boiled fat is used in order to represent the infected fat tissue. Hence, the antenna was implanted into the fat samples and the results revealed noticeable variations in the radiation characteristics between native and boiled fat. Different parameters of the proposed antenna including the reflection coefficient (S_11_), radiation patterns, gain, efficiency, and front-to-back ratio are investigated. The simulations showed that S_11_ parameter was − 12.4 dB in MICS band for the normal fat. On the other hand, the measured S_11_ values were − 12.3 dB for the native samples and − 9.9 dB for the boiled fat samples. To assert the variation in the biological characteristics of the boiled fat as compared to those of the native fat, diffuse optical measurements of the examined samples were investigated. Such variation in the light scattering and absorbance by the tissue is responsible for varying the S_11_ parameter for each case. The results have shown that the proposed design is a good candidate for detecting the change in biological tissue.

## Introduction

Biomedical antenna has been developed in 1960s by Medtronic, USA^1^ as a battery powered device. Nowadays, antenna in medical applications provides a virtual environment that has the ability to stimulate and monitor the function of various body organs^2^. The antenna wirelessly transfers information about glucose level, body temperature, pH, and cardiac pressure to a certain receiver (e.g., base-stations in medical centers) via links named “bio-telemetry”^3–5^. The design of the antenna depends on the industrial, scientific and medical (ISM) band for medical telemetry operations. Nonetheless, a recent medical implant communication service (MICS) band (402–405 MHz) has been allocated which is regulated by the United States federal communications commission and the European radio communications committee for bi-directional bio-telemetry operations^6^.

The MICS band permits low noise propagation through human body that makes it suitable for low power implanted device circuits^7^. The wavelength for free space is 74 cm in the med radio whereas it is around 12 cm in the ISM bands^8^. Accordingly, there is a need to miniaturize the antenna to be implanted inside the human body considering the biocompatibility properties and other related parameters such as gain, SAR, bandwidth, and directivity. Studying the miniaturized antenna and its radiation characteristics has started since decades. The size of the antenna was directly proportional to the radiation power factor, the total efficiency, and the antenna bandwidth^7^. Later on, different miniaturization techniques has been proposed including the use of high permittivity dielectric substrate, lengthening the current flow path on the patch surface, and inserting shorted pins between the ground and the patch plane. The latter results in developing antennas of relatively small effective size^9^.

Since the signal is highly attenuated within the human body, the near-field effect of antenna causes an increase in the temperature of neighboring tissues. Higher frequency operation also causes similar effect. This may be useful for some therapeutic applications yet shows a negative impact on communication^7^. Utilizing meandered antennas has solved such problem. The meandered structure can miniaturize the size of the antenna and keep the same radiation properties for an implantable antenna^8^.

Micro-strip (or patch) antennas have the advantages of being low cost, light weight, compact size and easy fabrication. However, a typical micro-strip antenna has a relatively narrow bandwidth^10^. Therefore, various techniques to enhance the bandwidth of patch antennas have been proposed including substrate-integrated suspended-line technique^11^, slotted patch^12,13^, adding stubs^14,15^, proximity coupled technique^16^, and cutting edges^17^. Also, the use of rectangular-shaped coplanar waveguide (CPW) fed printed antennas provides wider bandwidths necessary for wireless and wearable applications^18–21^. Additionally, to increase the gain of the antenna, several advancements are used such as artificial magnetic conductors (AMCs)^22^, metamaterials^23^ or a horn reflector^24^.

Various designs of wireless antennas were developed based on the use of rigid substrates such as FR4 as reported in^25–27^. Nevertheless, for implanting purpose, the design of efficient antennas requires the use of flexible substrates which have the ability to be incorporated with shapes of irregular surfaces^28^. Example of these flexible substrate are kapton^29^, liquid crystal polymer (LCP)^30^, Rogers RT/duroid^31^, cotton layer^32^, polyethylene terephthalate (PET) film^33^, and paper^34^. A flexible material such as Rogers ULTRALAM has been utilized for multiband conformal implantable antenna fabrication which is utilized in ingestible capsule endoscope and biotelemetry applications^35^. Moreover, antennas can be designed in different shapes including flat and capsule shapes. The capsule antennas are suitable for deep tissue implantation such as intestine, stomach and heart, while the flat type is used for skin (e.g., scalp) implantation^36,37^. Table 1 presents a comparison between the proposed antenna performance and the state-of-the-art designs.

**Table 1:** Performance comparison between the proposed antenna and the relevant literature.

Ref	Antenna dimension (mm^3^)	Resonant Freq. (GHz)	BW (MHz)	Gain (dBi)	SAR W/Kg	Test tissue
^35^	57	0.402	38.6	− 30.8	289	Minced pork
^38^	10 × 10 × 2.54	0.402	115	− 7	341	Pork
^39^	20.5 × 30 × 0.05	0.402	3.73	− 32	513.7	Deep tissue
^40^	7 × 6.5 × 0.377	0.402	36.8	− 30.5	588	Skin layer
^41^	22 × 23 × 1.27	0.402	30	− 36.7	832	Skin-mimicking material
^42^	14 × 7.5 × 0.5	0.405	64	− 40.85	665.35	Scalp (realistic human phantom)
This work	14 × 14 × 0.76	0.454	0.23	− 10	50	Fat

In the present paper, a micro-strip antenna is designed for monitoring adipose tissue (fat) at two different conditions (native and boiled). The antenna is designed in a simple double layered meandered structure. The different antenna parameters are evaluated at each tissue condition. The proposed antenna radiates near the MICS band for data transmission related to patient monitoring. Additionally, diffuse reflectance and transmittance of the examined samples were measured at four laser wavelengths (532, 660, 780 and 980 nm) using integrating sphere-based optical setup to emphasize the difference in tissue properties at each condition, which in turn results in varying the reflection coefficient (S_11_).

## Antenna design

### Design process

**Figure 1:**
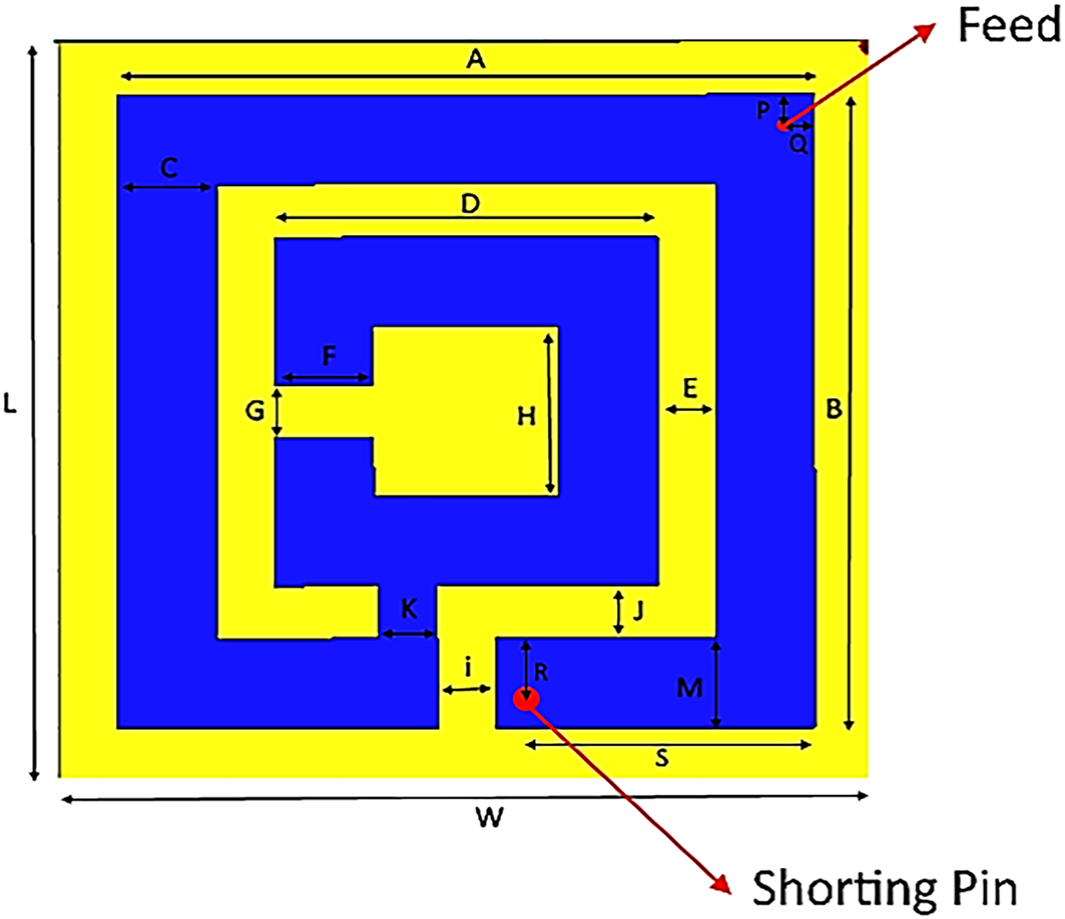
The proposed antenna configuration and dimensions.

A compact antenna of dimensions of 0.116 λ_0_ × 0.116 λ_0_ × 0.006 λ_0_ with respect to the resonant frequency is proposed in the current study. The simulations are performed using a commercial simulation software (High-Frequency Structure Simulator Technology (HFSS)). The design is applied on a dielectric substrate Rogers 3210 (dielectric constant = 10.5) to achieve the required characteristics. This substrate has a thickness of 0.76 mm and a loss tangent of 0.0027. It should be noted that the design is primarily based on the meander patch antenna. Figure 1 presents the meander patch antenna. Moreover, the dimensions of the proposed antenna are listed in Table 2.

**Table 2:** Dimensions of the proposed antenna (in mm).

A	B	C	D	E	F	G	H	i	J	K	L	M	h	W	P	Q	R	S
12	12	1.7	6.6	1	1.7	1	3.2	1	1	1	14	1.7	0.76	14	0.8	0.5	1.2	5

**Figure 2:**
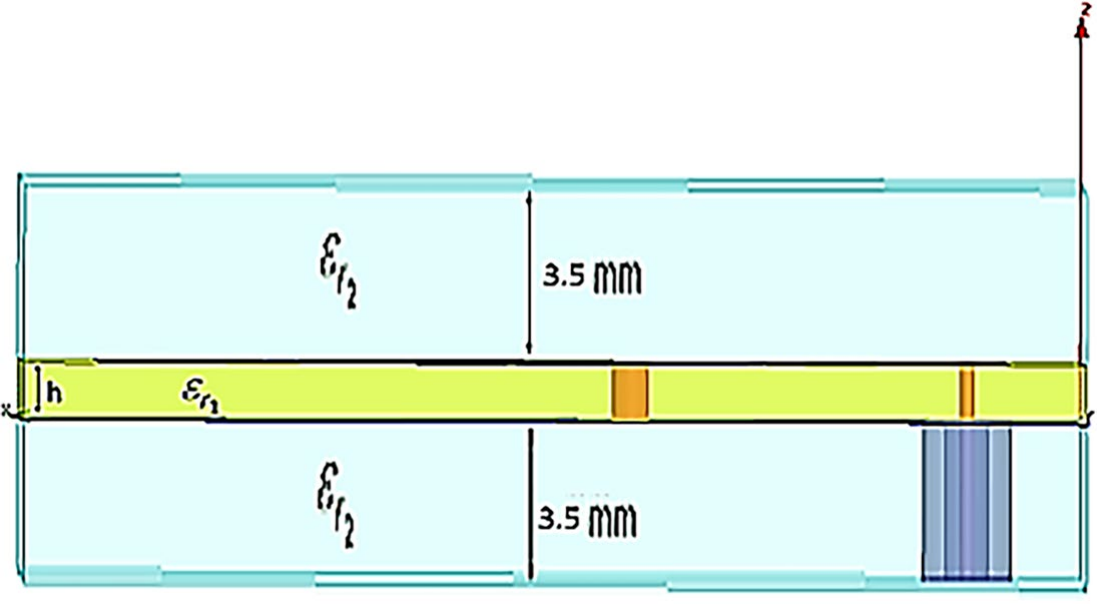
The simulation of the measurement setup in fat.

The first step in the design process is to identify the medium. The proposed antenna is designed to test fat tissue which has dispersive electromagnetic characteristics. The antenna design achieved the 50-Ω excitation in both cases. Figure 2 shows the simulation model in case of fat as a medium. As shown in Fig. 2, the antenna is sandwiched between two layers of fat. The preliminary simulation results for the native fat tissue shows that the resonance frequency achieved by our proposed antenna is 513 MHz.

### Design approach

**Figure 3:**
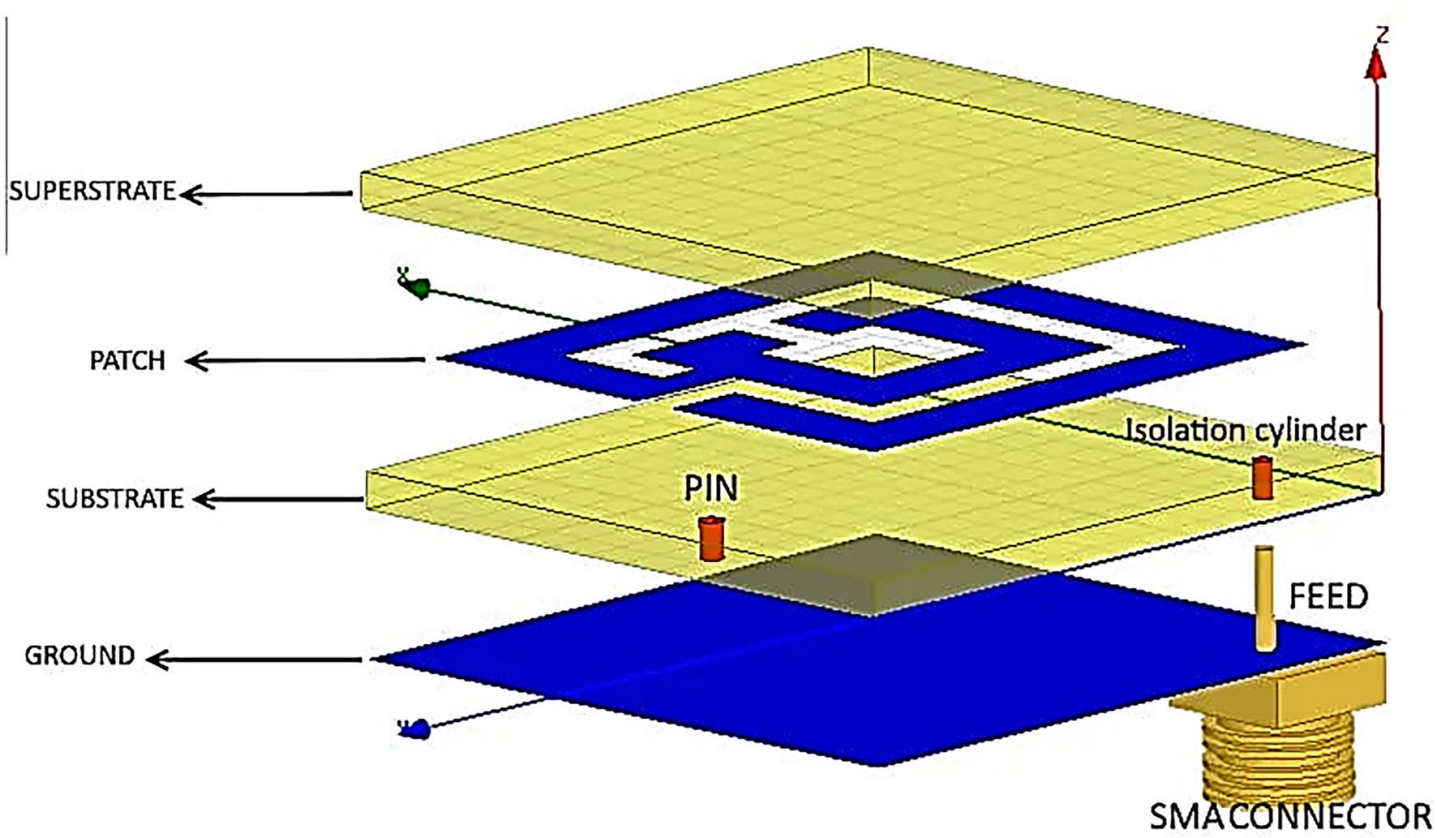
The common design approach of meander antenna.

The proposed antenna consists of three layers. The first layer is a ground sheet connected with the patch through the pin. The second layer is the substrate which is divided into two layers that sandwiches the patch (the third layer) between them (see Fig. 3).

The patch layer of the antenna is attached at the top right corner with SMA connector pin. This layer also includes meander tailored resonators and a capacitive coupler gap^43^. The proposed design aims to elongate the current path. The meander structure is bent to decrease the length and increase stiffness of the antenna. A superstrate is usually added to avoid the short circuit between patch and substrate. Therefore, this eliminates the patch to function as a radiating element. Nevertheless, in our proposed design, there is no need to use a superstrate due to the poor conductivity of fat material (0.102 S/m). Table 3 represents fat parameters in the resonance frequency. The manufacturing and measurements were performed in the National Telecommunication Institute (NTI), Cairo, Egypt.

**Table 3:** Fats parameters in the resonance frequency.

Tissue	Conductivity	Relative permittivity	Loss tangent	Wavelength	Penetration depth
Fat	0.10235 S/m	5.2853	0.14503	0.054193 m	0.11956 m

Indeed, the microstructural and macroscopic properties of boiled fat are relatively different from the native one^44^. Once the fat is heated, adipose tissue (fat) is liberated from the adipocytes (a cell in connective tissue that is specialized for the fat storage) and its oxidation increases^45^. Accordingly, the electrical conductivity of the fat tissue increases as the temperature increases while decreases if its structure changes^46^.

### The current distribution

**Figure 4:**
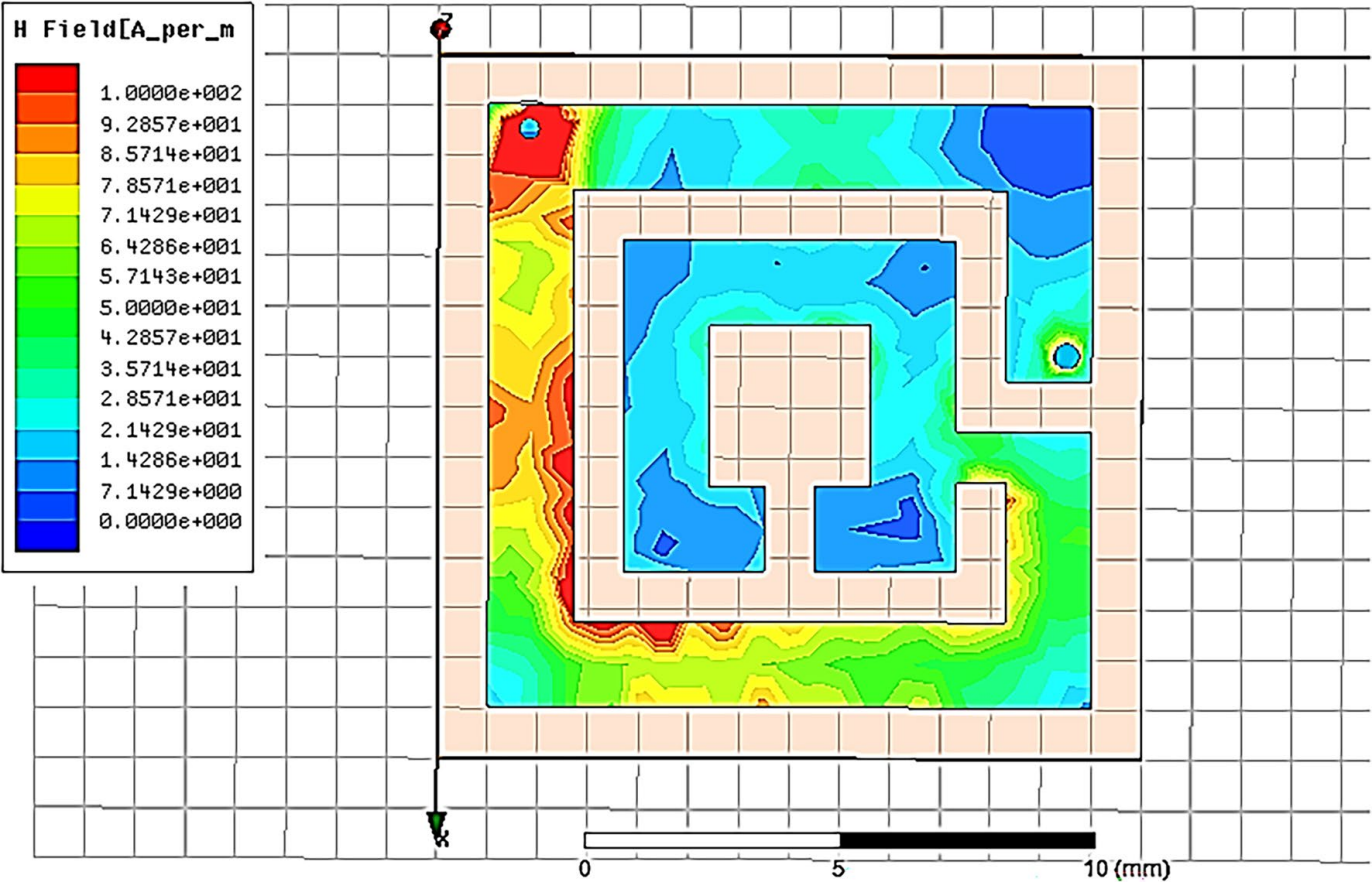
Current distribution of the proposed antenna at MICS band.

To illustrate the mechanism of operation, the surface current distribution for the proposed antenna is presented in Fig. 4. The current distribution demonstrates that the antenna structure is responsible for producing l dip at 513 MHz as the current concentration along the antenna length. The current concentrates along the antenna width, patch, and pin. The intensive current indicated by red and a null represented by blue. The figure also illustrates the effective area at which the proposed antenna resonates at the certain frequency.

## Diffuse optical measurements

**Figure 5:**
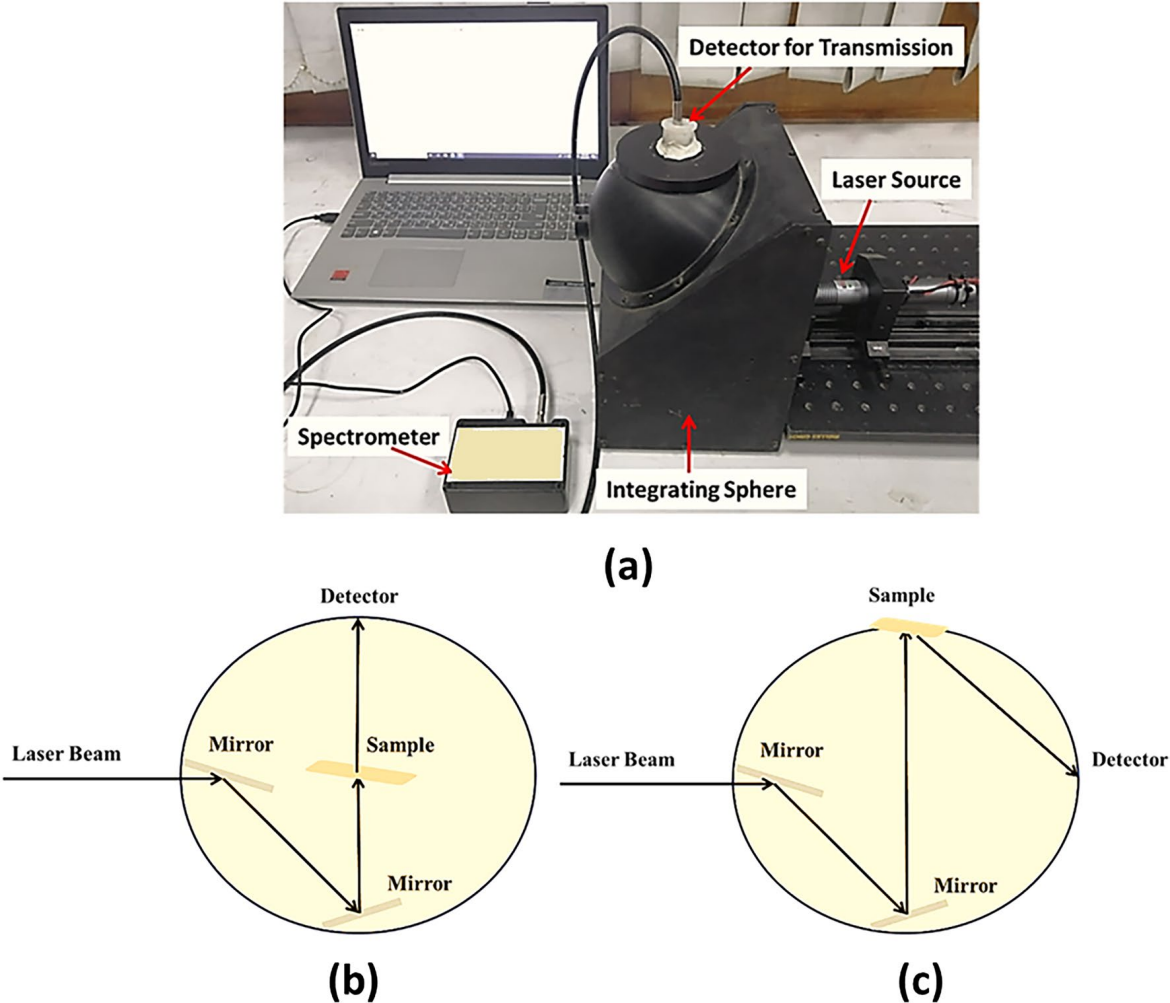
(**a**) The implemented optical setup using integrating sphere, (**b**) schematic of the transmission mode, and (**c**) schematic of the reflection mode.

The optical transmission and reflection resulting from light interaction with biological tissue is named as diffuse because of the multiple scattering events affected the light propagation in tissues^47^. The tissue diffuse reflectance/transmittance is a representative parameter for each tissue, where they vary according to the tissue condition. Therefore, they can be used in tissue differentiation processes^48,49^. Integrating sphere is a very common device that is used for the ex-vivo measurement of the tissue diffuse reflectance and transmittance^50,51^. It is a hollow spherical cavity coated with highly reflective material to ensure reflection of the whole entering light. In our experiments, Barium sulfate coated mcPHERSON integrating sphere has been used to measure diffuse reflectance/transmittance of the native and boiled fat samples. A photo of the experimental setup is presented in Fig. 5a.

A typical integrating sphere has multiple ports and locations for placing the sample under investigation. Furthermore, a light detector is used where its location is dependent on the required measurement (tissue transmittance or reflectance) as demonstrated in Fig. 5b,c, respectively. The tissue’s detected diffuse light is then delivered to a compact spectrometer that is connected to a computer for data analysis and processing. In the present work, a digital fiber spectrometer (STDFSM, Touptek Photonics Co. Ltd, Zhejiang China) integrated with a detector (Toshiba TCD1304AP linear CCD array) has been used.

### Collecting fat samples

Ex-vivo bovine intramuscular fat (adipose tissue) samples have been collected from different butcher shops. There is no direct contact with alive animals in the present study. Therefore, no specific ethical approval is required. The sources of the utilized fat samples (bovines) were already slaughtered for commercial food production and the fat tissues can be considered as disposals or remains.

## Results and discussion

**Figure 6:**
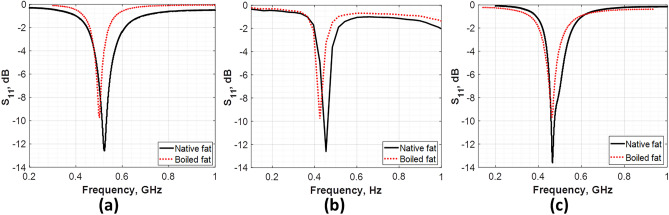
The measured reflection coefficient (S11) of the three fabricated antennas for native and boiled fat tissues.

To evaluate the proposed design, a Rohde&Schwarz ZVB 20 (Vector Network Analyzer, 10 MHz–20 GHz) is used to test the antenna. As previously mentioned, the fat is examined in this study in two cases; normal and infected (the infected was represented by the boiled tissue). For better profess, three similar antennas with the same design and material were fabricated and measured. Figure 6 shows the measured reflection coefficient of each antenna at the two examined tissue conditions.

**Figure 7:**
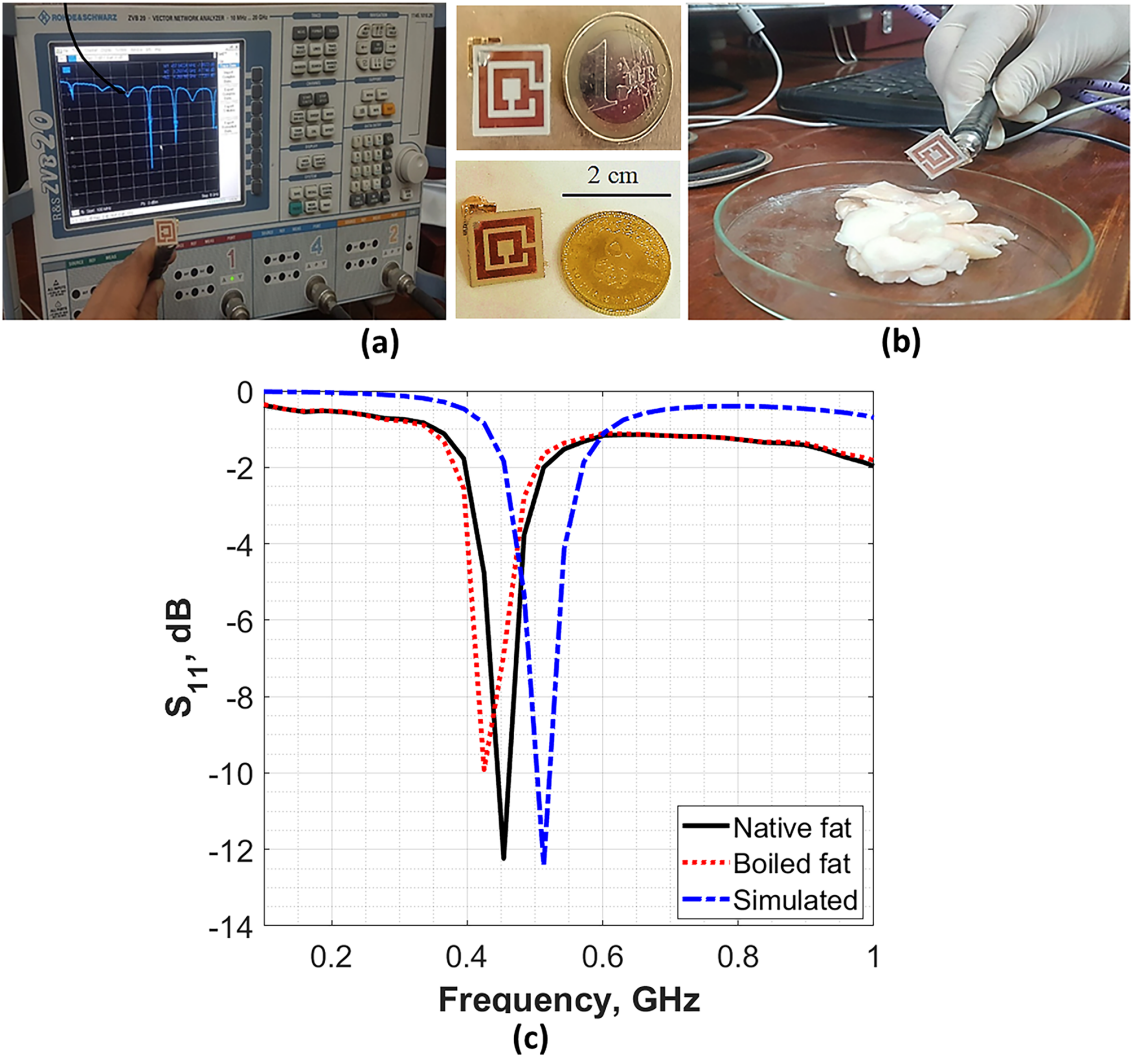
(**a**) A photograph of the fabricated antennas, (**b**) a photograph of the antenna with studied fat sample, (**c**) the S_11_ parameters for simulation, normal and boiled fat.

Photographs of the fabricated antennas before and after immersed in the fat sample is presented in Fig. 7a,b respectively. Moreover, a comparison between the simulated and average measured reflection coefficient (S_11_) for the three antennas is presented in Fig. 7c.

Figure 7c illustrates the S_11_ parameter for the three cases; simulations, native tissue and infected tissue (boiled). In the simulations, S_11_ was − 12.4 dB at 0.513 GHz. However, in the case of native fat the S_11_ was − 12.3 dB. In case of infected fat S_11_ changed to − 9.9 dB. In the measurements, both native and infected fat exhibit a frequency shift to the left with respect to that of the simulated value (i.e., 0.454 GHz for the native fat and 0.425 GHz for the infected fat). The observed frequency shift in the three cases is attributed to the non-uniform thickness (structure) of the examined fat tissue samples at the different conditions (native and boiled samples). In addition, the possible fabrication tolerance may lead to the same effect. Table 4 summarizes the results of the proposed antenna.

**Table 4:** Summary of the simulated and measured antenna results.

	Validation	Operating frequency (GHz)	Reflection coefficient, S11 (dB)	− 3- dB Bandwidth (MHz)
Simulated	Native fat	0.513	− 12.4	0.20
Measured	Native fat	0.454	− 12.3 ± 0.3	0.19 ± 0.02
	Boiled fat	0.425	− 9.9 ± 0.2	0.17 ± 0.01

### The radiation patterns

**Figure 8:**
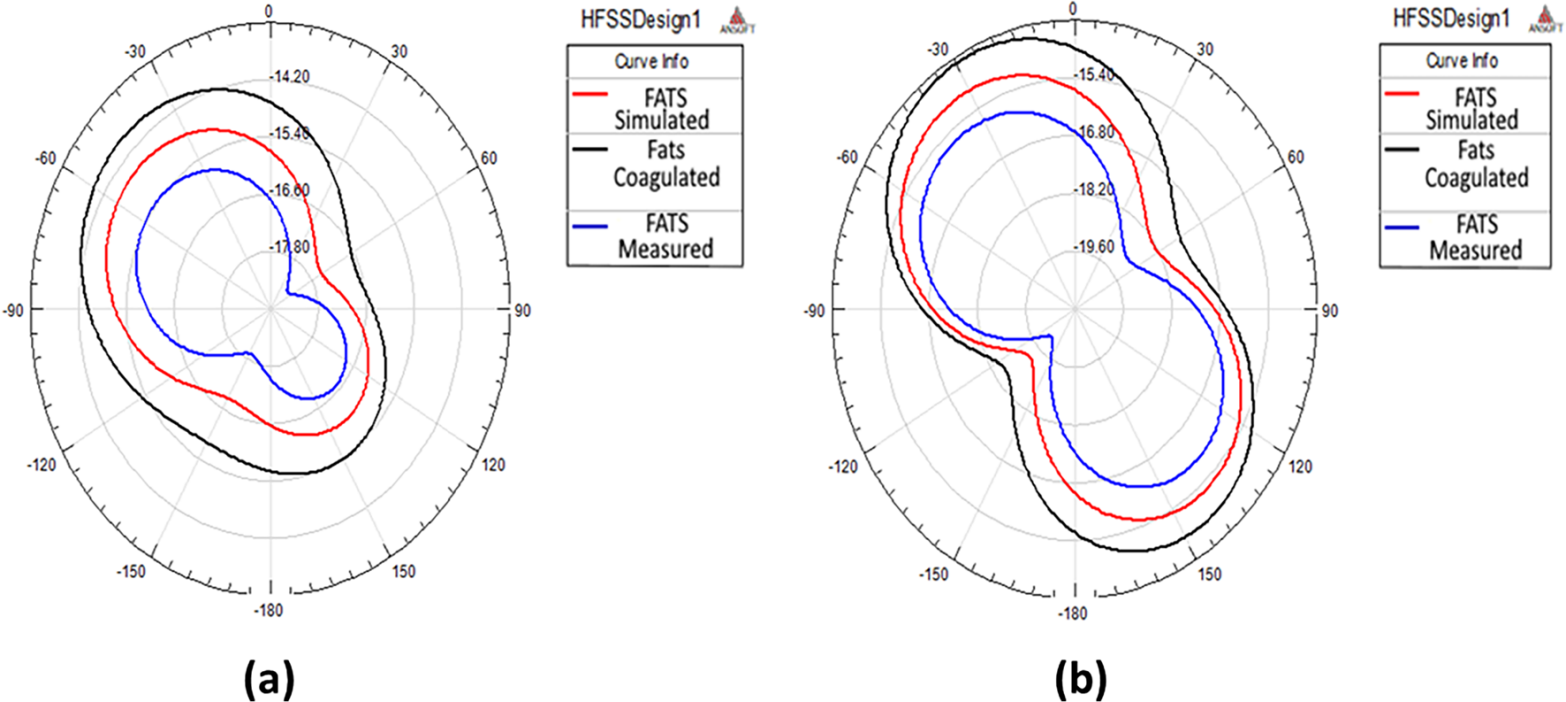
The Radiation pattern (**a**) E-plane at φ = 0 and (**b**) H-plane at φ = 90°.

The E-plane of the radiation pattern of the proposed antenna (E_θ_ at φ = 0°) and the H-plane radiation pattern (E_θ_ at φ = 90°) is presented in Fig. 8.

The E-plane radiation patterns show that the proposed antenna radiates omnidirectional in case of simulated and boiled tissue and bi-directional in case of normal fat. However, the H-plane patterns reveal that the antenna radiates bi-directional in the three cases. It is worth noting that the model does not take into account the probe location. It is also assumes that the dielectric material of the substrate is truncated and does not cover the ground plane beyond the edges of the patch which may be the cause of the discrepancies between the two patterns.

**Figure 9:**
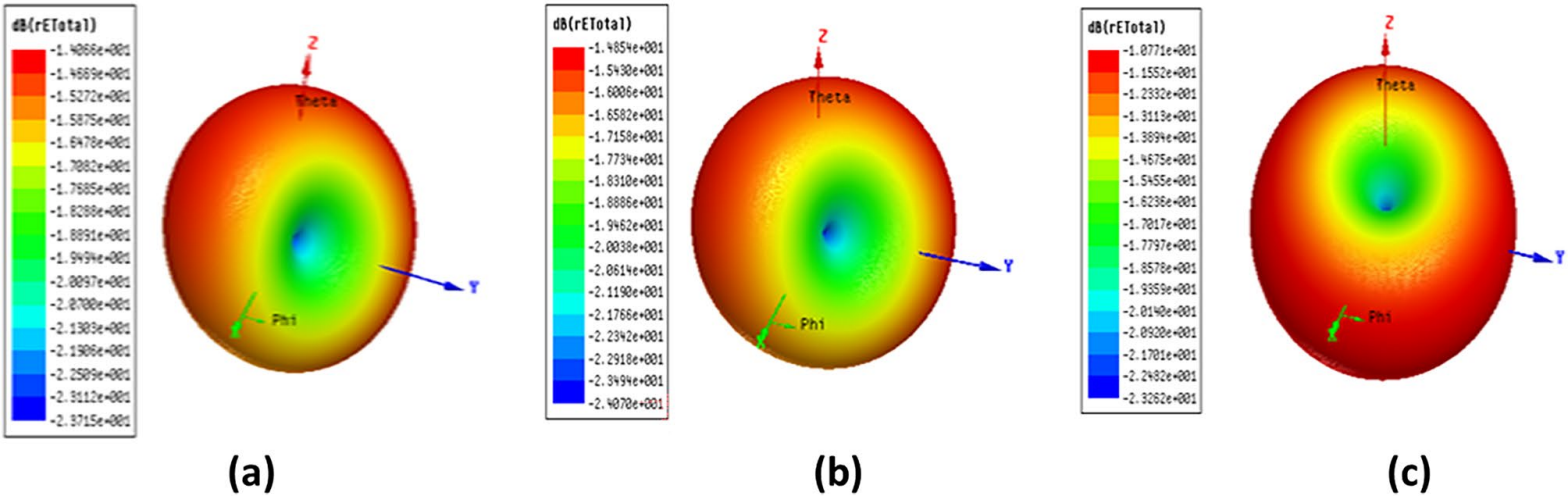
The 3-D radiation patterns for the proposed antenna (**a**) simulation, (**b**) normal fat and (**c**) boiled fat.

The 3-D radiation patterns for the proposed antenna at the resonance frequency for the three configurations; simulated, normal and boiled fat are illustrated in Fig. 9a–c, respectively.

### The gain

**Figure 10:**
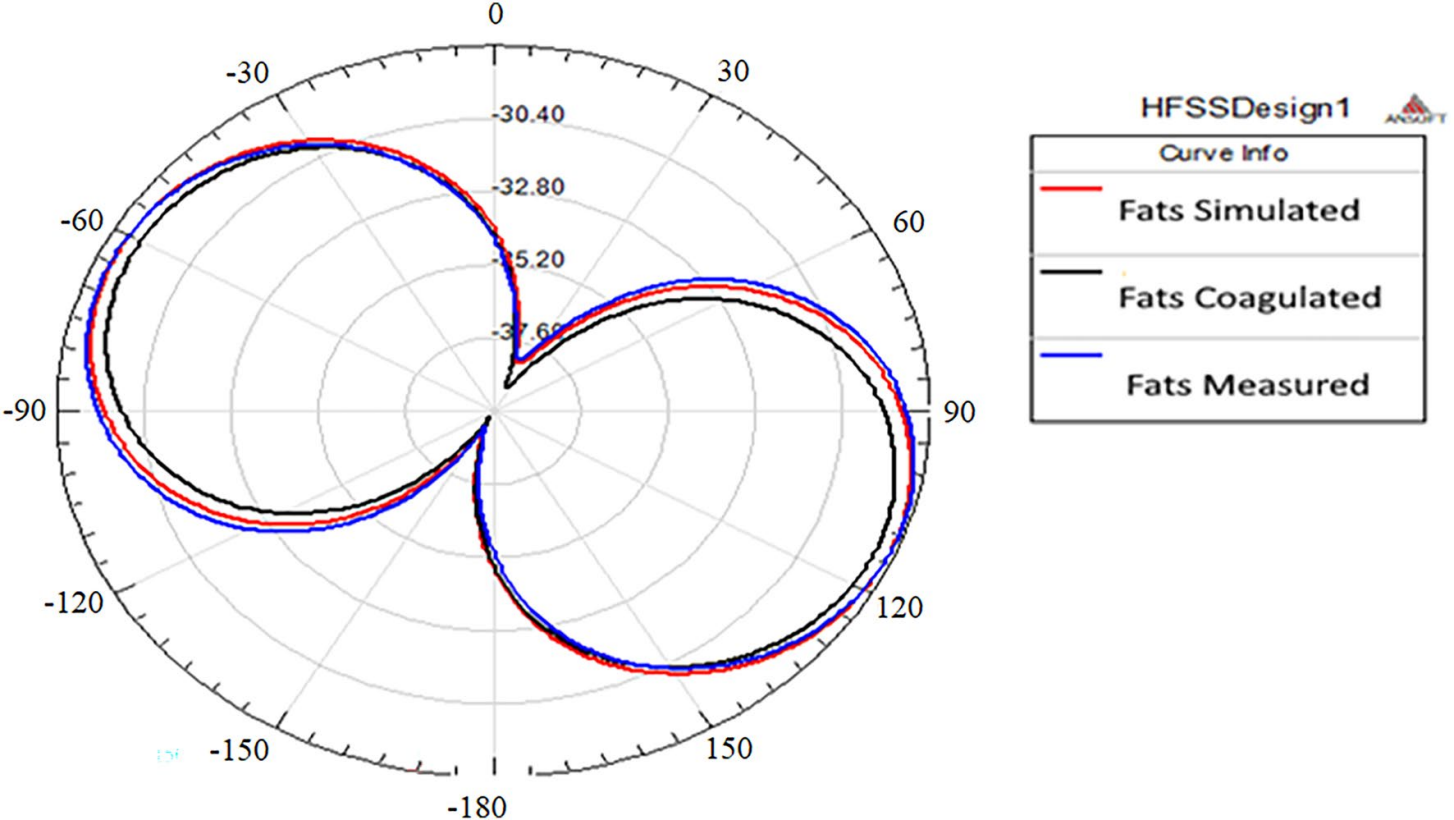
The gain of the proposed antenna.

Figure 10 shows the gain of the proposed antenna. The maximum obtained gain in the MICS band was about − 10 dB. This is due to limitations in the measurement process in addition to the degenerative effect of the SMA connector due to small size of the antenna.

### SAR

The electromagnetic parameters of the human tissue are given in Table 5. The calculation formula of SAR value is presented as follows:

1 $$ SAR = \frac{{\sigma E^{2} }}{\rho } $$

where σ is conductivity, E is the electric field intensity, and ρ is the mass density^52^.

**Table 5:** The electromagnetic parameters of the human tissue.

Tissue	ε_r_	Conductivity (S/m)	Density (kg/m^3^)	Thickness (mm)
Skin	37.95	1.49	1001	2
Fat	5.27	0.10235	900	5
Muscle	52.67	1.77	1006	20
Bone	18.49	0.82	1008	13

**Figure 11:**
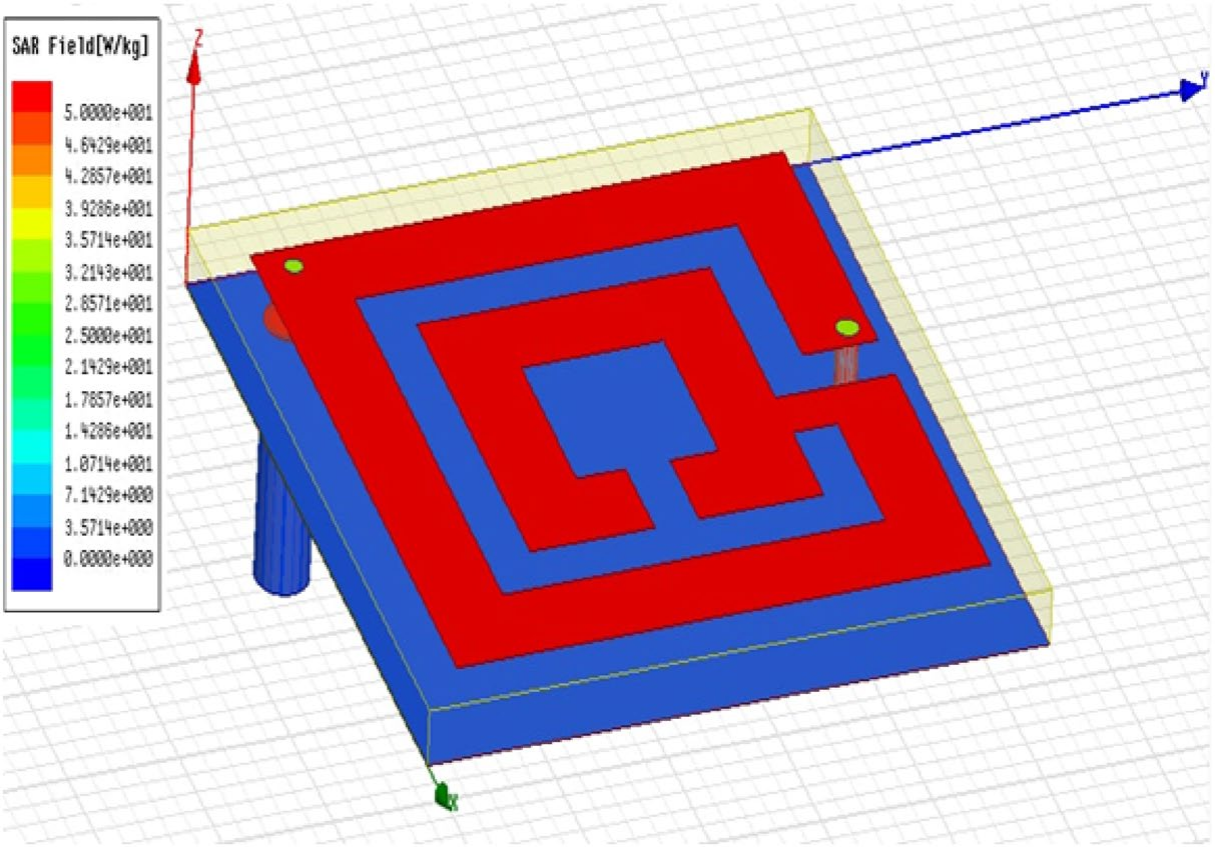
Simulated SAR of the proposed antenna.

The average SAR value of the proposed antenna when implanted into the fat tissue is presented in Fig. 11. The maximum SAR of the 10 g is 50 W/Kg. Due to the reflection characteristics, the obtained SAR enhances the main lobe but it also reduces the back lobe. This means that only a small amount of electromagnetic wave energy is radiated to the fat tissue. Therefore, the antenna has good forward radiation and reasonable SAR value.

### Total efficiency and front to back ratio

**Figure 12:**
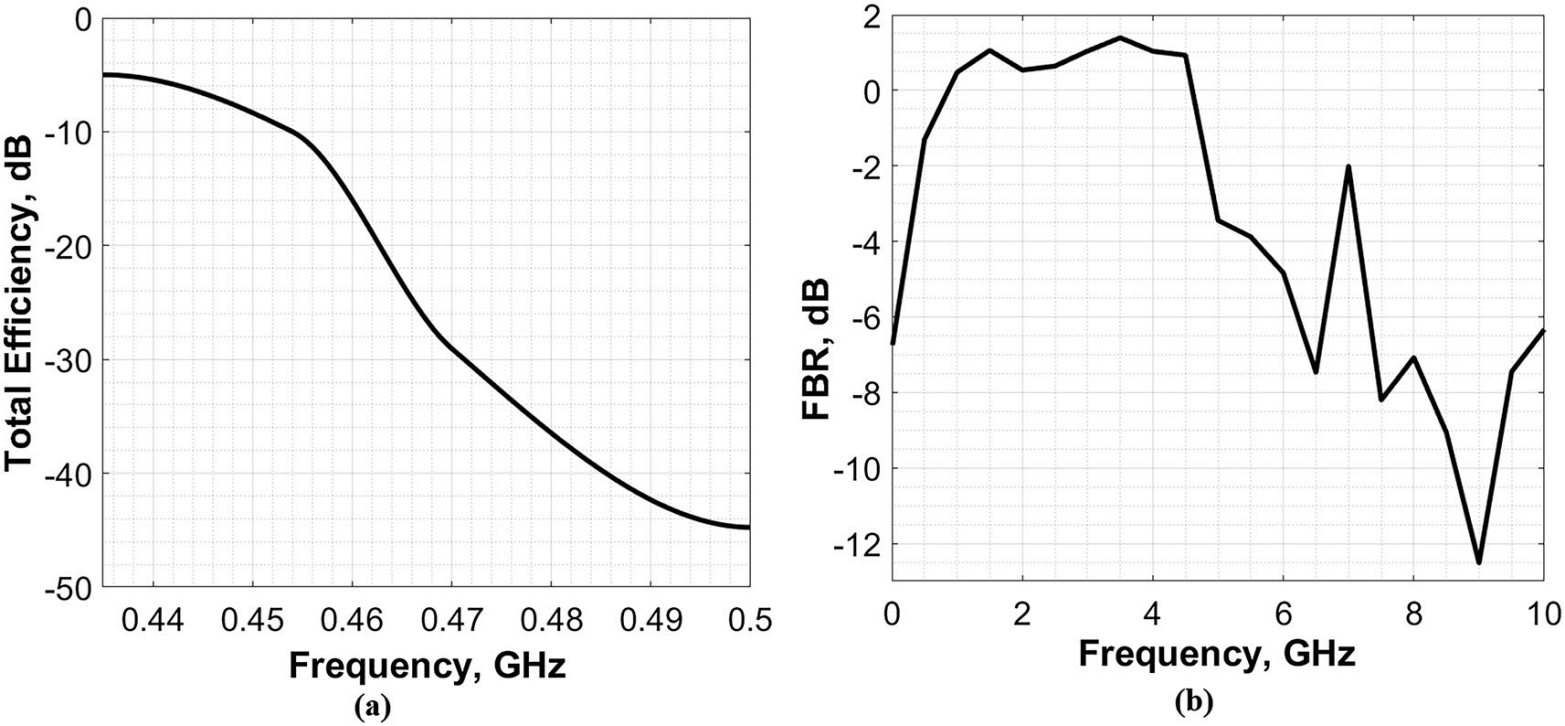
The simulated (**a**) total efficiency, and (**b**) front to back ratio of the proposed antenna.

The simulated total efficiency and front to back ratio “FBR” versus frequency of the proposed antenna are presented in Fig. 12a,b, respectively.

As shown in Fig. 12a, the total efficiency of the antenna is almost − 10 dB at the operational frequency. In addition, a relatively high FBR is obtained at the resonance frequency of the antenna (see Fig. 12b).

### Diffuse optical measurements

**Figure 13:**
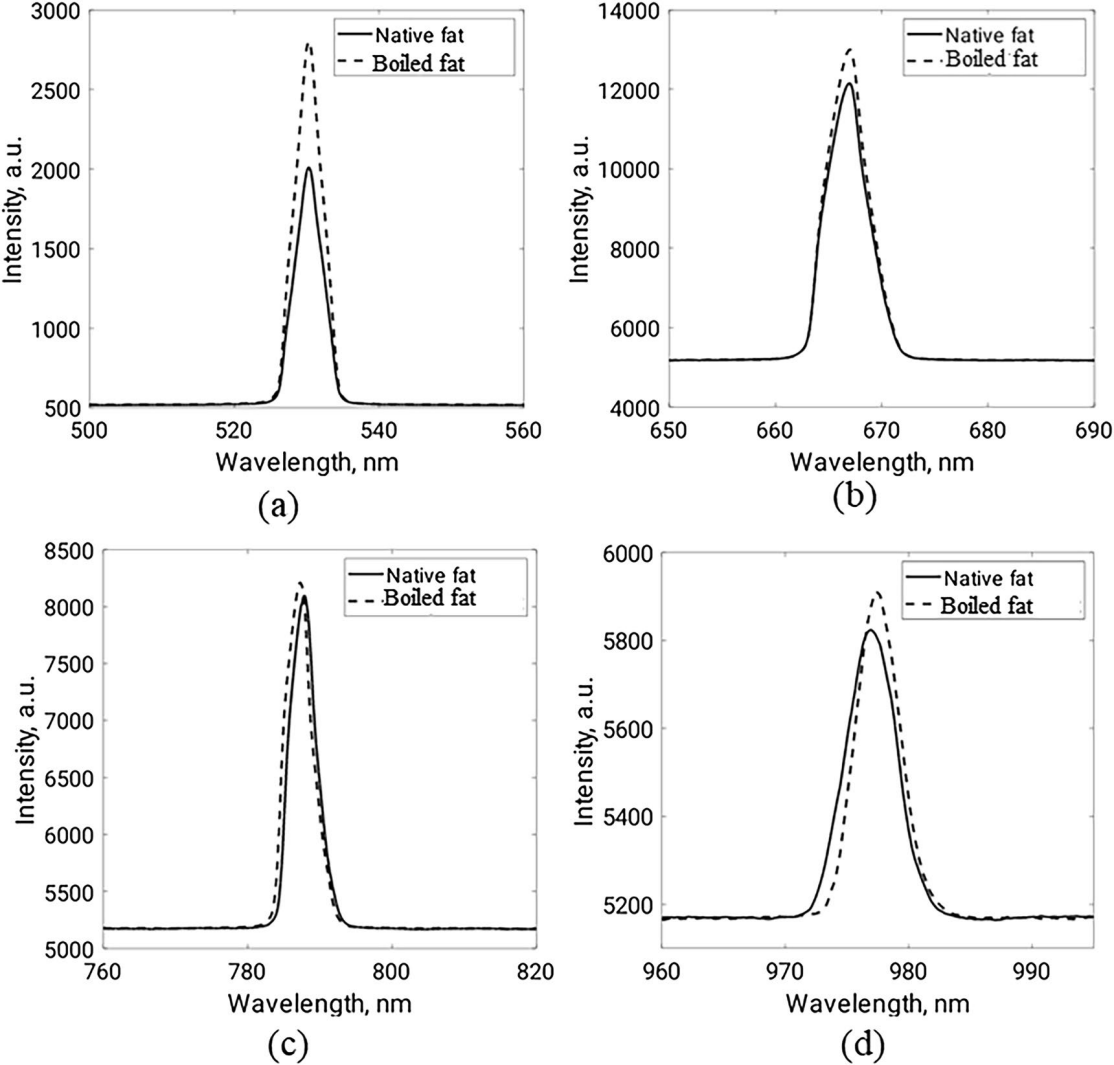
Diffuse reflected light of fat samples at (**a**) 532 nm, (**b**) 660 nm, (**c**) 780 nm, and (**d**) 980 nm.

**Figure 14:**
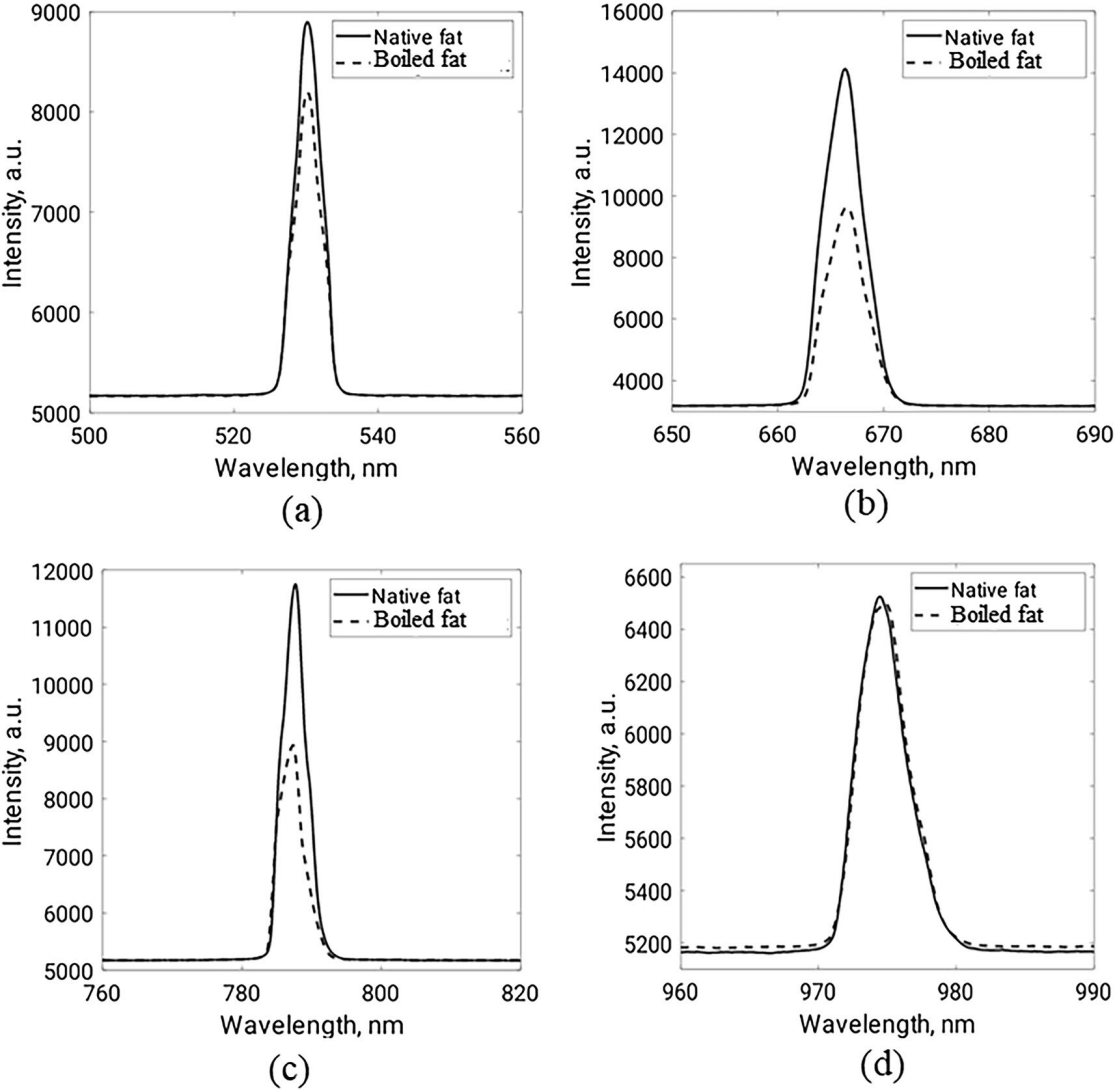
Total transmitted light of fat samples at (**a**) 532 nm, (**b**) 660 nm, (**c**) 780 nm, and (**d**) 980 nm.

Tissue reflectance is the ratio between the reflected light intensity and the incident light intensity, while the transmittance is defined as the ratio between the transmitted light intensity and the incident light intensity. Accordingly, the variations in the reflected light intensity and transmitted light intensity of the fat samples (native and boiled) at each wavelength are presented in Figs. 13 and 14, respectively. For each sample, the experimental measurements have been obtained five times and the average values are plotted.

As shown in Fig. 13, the diffuse reflectance of the boiled fat tissues is less than the native tissues at the four studied wavelengths. On the other hand, the transmittance measurements show the opposite behavior (Fig. 14). Such results are reasonable as some cellular and intracellular characteristics (such as protein denaturation, hyalinization of collagen, and cell membrane rupture) of fat tissues are supposed to be altered after boiling^53^. These characteristics have a great effect on tissue scattering properties and hence affect light distribution and propagation^54^.

## Conclusions

A miniaturized multilayer antenna was designed as a diagnostic tool for differentiating native and infected adipose tissue (ex-vivo bovine fat). An additional substrate was added to the design to enable the antenna to test high conductive tissues. The antenna resonated at 454 MHz (near to the MICS band), which makes it applicable for the short range medical applications. Although the resonant frequency of the proposed antenna does not lay exactly in the MICS band (402–405 MHz), the measured reflection coefficient (S_11_) showed a considerable change at the different conditions of the tissue. Additionally, the measured results of the proposed antenna showed an acceptable agreement with the simulation results. Moreover, the E- and H-field radiation patterns asserted the same conclusions. The antenna gain was − 10 dB in the three cases. Furthermore, the diffuse reflectance and transmittance of the studied tissue samples were measured to confirm the variation in the optical properties between native and infected samples which was also demonstrated in the obtained S_11_ parameter at each case. Compared with the previous literature, the proposed antenna has relatively small dimensions. For future investigations, the proposed antenna will be tested on other tissues with high conductivity. Additionally, multiple antennas with different designs using various Rogers will be considered.

## Data Availability

The datasets used and/or analyzed during the current study available from the corresponding author on reasonable request.
